# Antibiotics Treatment Modulates Microglia–Synapses Interaction

**DOI:** 10.3390/cells10102648

**Published:** 2021-10-04

**Authors:** Federica Cordella, Caterina Sanchini, Maria Rosito, Laura Ferrucci, Natalia Pediconi, Barbara Cortese, Francesca Guerrieri, Giuseppe Rubens Pascucci, Fabrizio Antonangeli, Giovanna Peruzzi, Maria Giubettini, Bernadette Basilico, Francesca Pagani, Alfonso Grimaldi, Giuseppina D’Alessandro, Cristina Limatola, Davide Ragozzino, Silvia Di Angelantonio

**Affiliations:** 1 Department of Physiology and Pharmacology “V. Erspamer”, Sapienza University, 00185 Rome, Italy; federica.cordella@uniroma1.it (F.C.); caterina.sanchini@uniroma1.it (C.S.); laura.ferrucci@uniroma1.it (L.F.); bernadette.basilico@ist.ac.at (B.B.); giuseppina.dalessandro@uniroma1.it (G.D.); davide.ragozzino@uniroma1.it (D.R.); 2 Center for Life Nano- & Neuro-Science, Fondazione Istituto Italiano di Tecnologia (IIT), 00161 Rome, Italy; natalia.pediconi@iit.it (N.P.); giovanna.peruzzi@iit.it (G.P.); pagani.f@hotmail.com (F.P.); alfonso.grimaldi@uniroma2.it (A.G.); 3 National Research Council-Nanotechnology Institute, 00185 Rome, Italy; barbara.cortese@nanotec.cnr.it; 4 Cancer Research Center of Lyon (CRCL), UMR Inserm U1052/CNRS 5286, 69373 Lyon, France; francesca.guerrieri@inserm.fr (F.G.); giuseppe92312@gmail.com (G.R.P.); 5 Department of Molecular Medicine, Laboratory Affiliated to Istituto Pasteur Italia, Sapienza University, 00185 Rome, Italy; fabrizio.antonangeli@uniroma1.it; 6 CrestOptics S.p.A., Via di Torre Rossa 66, 00165 Rome, Italy; giubettini@crestoptics.com; 7 Department of Physiology and Pharmacology, Laboratory Affiliated to Istituto Pasteur Italia, Sapienza University, 00185 Rome, Italy; cristina.limatola@uniroma1.it; 8 IRCCS Neuromed, Via Atinese 18, 86077 Pozzilli, Italy; 9 Santa Lucia Foundation, European Center for Brain Research, 00143 Rome, Italy

**Keywords:** microglia, gut–brain axis, antibiotics, glutamatergic synapses, hippocampus, patch clamp, hippocampal slices, CX3CL1/CX3CR1

## Abstract

‘Dysbiosis’ of the adult gut microbiota, in response to challenges such as infection, altered diet, stress, and antibiotics treatment has been recently linked to pathological alteration of brain function and behavior. Moreover, gut microbiota composition constantly controls microglia maturation, as revealed by morphological observations and gene expression analysis. However, it is unclear whether microglia functional properties and crosstalk with neurons, known to shape and modulate synaptic development and function, are influenced by the gut microbiota. Here, we investigated how antibiotic-mediated alteration of the gut microbiota influences microglial and neuronal functions in adult mice hippocampus. Hippocampal microglia from adult mice treated with oral antibiotics exhibited increased microglia density, altered basal patrolling activity, and impaired process rearrangement in response to damage. Patch clamp recordings at CA3-CA1 synapses revealed that antibiotics treatment alters neuronal functions, reducing spontaneous postsynaptic glutamatergic currents and decreasing synaptic connectivity, without reducing dendritic spines density. Antibiotics treatment was unable to modulate synaptic function in CX3CR1-deficient mice, pointing to an involvement of microglia–neuron crosstalk through the CX3CL1/CX3CR1 axis in the effect of dysbiosis on neuronal functions. Together, our findings show that antibiotic alteration of gut microbiota impairs synaptic efficacy, suggesting that CX3CL1/CX3CR1 signaling supporting microglia is a major player in in the gut–brain axis, and in particular in the gut microbiota-to-neuron communication pathway.

## 1. Introduction

The influence of the gut–brain axis in maintaining brain homeostasis has long been appreciated. However, in past years the role of the microbiota has emerged as one of the key regulators of gut–brain function, leading to the definition of a novel microbiota–gut–brain axis (MGBA; [1]). This axis, and in particular the gut microbiota composition, has been linked to the biological and physiological basis of psychiatric, neurodevelopmental, age-related, and neurodegenerative disorders [1]. The microbiota–brain communication encompasses several possible routes, such as the immune system, the tryptophan metabolism, the vagus nerve and the enteric nervous system, involving microbial metabolites such as short-chain fatty acids, branched chain amino acids, and peptidoglycans [2]. The manipulation of gut microbiota in animal models has become a paramount paradigm for disclosure of the causative factors linking the microbiota composition to the regulation of neural and cognitive processes. In addition, ongoing clinical trials are investigating the role of MBGA manipulation for the treatment of brain disorders (Clinical trials.gov Identifier: NCT03237078; NCT04366401 studies). During life, many factors can influence microbiota composition, including infection, mode of birth delivery, use of antibiotic (ABX) medications, nutritional supplements, environmental stressors, host genetics and aging. Moreover, microbiota and its metabolites have been suggested to be involved in the modulation of brain functions, such as emotional behaviors [3] stress-related responsiveness [4], pain [5], and food intake [6]. Consequently, alterations of the ‘‘healthy” microbiota, referred to as dysbiosis, might drive functional and behavioral changes in animals and humans [7,8].

In this context, preclinical studies have demonstrated that ABX administration has long-lasting effects on the brain, the spinal cord, and the enteric nervous system [9]. Indeed, ABX are known to profoundly alter gut microbiota, possibly resulting in detrimental effects on brain function and behavior, such as memory impairment in object recognition associated with changes in the expression of related signaling molecules (i.e., BDNF, GRIN2B, 5-HT transporter, and NPY) [10,11]. Similarly, chronic long-term ABX treatment was found to induce memory deficits and to decrease hippocampal neurogenesis in adult mice [12,13], while acute treatments were ineffective in rats’ early life [14]. In addition, microbiota depletion due to ABX has been shown to impact stress-related behaviors, although the mechanism is still not clear [10,15,16]. 

Despite the huge amount of data pointing to the role of MGBA in modulating brain functions, there is an urgent need to understand the intricate processes and the cellular and molecular events involved. A possible mechanism linking MGBA and neuronal functions arises from the data showing that microbiota composition constantly controls microglia maturation [17]. In germ-free (GF) mice, microglia display an immature phenotype which can also be observed after four weeks of an ABX cocktail treatment of adult microbial colonized mice [17]. The reported microbiota modulation of microglia phenotype may underlie the effect of MGBA on brain function. 

Microglia (the CNS tissue macrophages) are crucial not only for the maintenance of brain homeostasis during development and adulthood, but also exert a profound effect on neurons, refining the neuronal network in physiological and pathological conditions, both directly through physical contacts or soluble factors release [18,19,20] and indirectly, modulating astrocytic beneficial or detrimental activity [21]. One of the key elements in the microglia–neuron crosstalk, deeply linked to the synaptic refinement and modulation, is the CX3CL1/CX3CR1 axis. Indeed, the disruption of this neuron–microglia signaling causes several alterations in brain connectivity [22] and cognitive functions [23] associated with an impairment in glutamatergic synaptic transmission [22,23,24,25,26]. These effects have been generally ascribed to the roles exerted by microglia during brain development, due to the ability of these cells to foster synaptic pruning [24], likely by contacting and phagocyting synaptic elements [19,27,28]. 

Given the impact of microbiota composition on microglia signature, and the role of microglia in tuning synaptic transmission, we explored the possibility that microglia, orchestrating the bidirectional crosstalk between the gut and the brain, might be the missing key element in the MGBA modulation of neuronal functions. For this purpose, we altered gut microbiota composition, treating mice with two non-absorbable ABX, and we evaluated the impact of two weeks of treatment on microglia and synaptic function. We demonstrated that ABX treatment profoundly affects the ability of microglia in monitoring brain parenchyma homeostasis and impairs the efficacy of hippocampal glutamatergic synaptic transmission. In addition, we showed that ABX did not alter glutamatergic function in CX3CR1-deficient mice, highlighting the involvement of the neuron to microglia CX3CL1/CR3CR1 axis in the microbiota-to-neuron communication pathway.

## 2. Materials and Methods

### 2.1. Animals

All procedures performed using laboratory animals were in accordance with the Italian and European guidelines and were approved by the Italian Ministry of Health in accordance with the guidelines on the ethical use of animals from the European Communities Council Directive of September 20, 2010 (2010/63/UE). All efforts were made to minimize suffering and number of animals used. Mice were housed in standard cages in a group of a maximum of 5 animals, with light–dark cycles of 12 h at 22±2 °C. Mice were divided into two experimental groups, control (CTRL) and antibiotic-treated (ABX). To avoid stress induced by oral gavage [29], ABX were administered in the drinking water and bottles were changed every second day. Both groups had sterile food and water ad libitum. Gentamicin (Gibco 15750037) and Vancomycin (Sigma V2002-1G), 0.5 mg/mL were administered starting from P28 for two weeks, 3 times a week, using sterile water feeders in a mix containing 50% sterile water and 50% sterile water plus sugar. The dose of antibiotics was adjusted according to the mean volume of water consumed on each day per mouse. Water was autoclaved and water intake was monitored daily. CTRL mice received only water solution (50% sterile water and 50% sterile water plus sugar) for two weeks. The ABX treatment was performed as in D’Alessandro et al., 2020 in the same animal facility, and with the same conditions. Mice were sacrificed at P40. 

For electrophysiological and time-lapse recordings, Cx3cr1^+/gfp^ and Cx3cr1^gfp/gfp^ mice were used; Cx3cr1^gfp/gfp^ mice were purchased from The Jackson Laboratory company (B6.129P2(Cg)-Cx3cr1tm1Litt/J); the colony was established in our animal facility, and progenitors were bred to C57BL6J to obtain Cx3cxr1^+/gfp^ mice as we previously reported [30]. Wild type C57BL-6J were purchased from Charles River and used for Nanostring and RT-PCR analysis. Thy1::EGFP-M21 mice, used for spine density analysis, were purchased from The Jackson Laboratory company. All experiments were performed on male mice.

### 2.2. Electrophysiological Recordings

Acute hippocampal slices were obtained from Cx3cxr1^+/gfp^ and Cx3cr1^gfp/gfp^ mice sacrificed at P40. Mice were decapitated under halothane anesthesia (Sigma Aldrich, Co., St. Louis, MO, USA). Whole brains were removed from the skull and rapidly placed for 10 min in ice-cold artificial cerebrospinal fluid (ACSF) containing (in mM): KCl 2.5, CaCl_2_ 2.4, MgCl_2_ 1.2, NaHSO_4_ 1.2, glucose 11, NaHCO_3_ 26 and glycerol 250 (Sigma Aldrich), 300 mOsm. ACSF was under continuous oxygenation (95% O_2_ and 5% CO_2_) to maintain the physiological pH. Horizontal 250-µm-thick slices were cut at 4 °C using a Ted Pella vibratome and placed in a chamber filled with oxygenated ACSF containing (in mM): NaCl 125, KCl 2.5, CaCl_2_ 2, MgCl_2_ 1, NaHSO_4_ 1.2, NaHCO_3_ 26 and glucose 10, 300 mOsm. Slices were left to recover for at least 1h at room temperature until use (24 ± 1 °C). All the experiments were performed at room temperature on slices submerged in ACSF and perfused with the same solution in the recording chamber. Spontaneous currents (sPSC) and excitatory postsynaptic currents were recorded from CA1 pyramidal neurons at −70 mV, using a patch clamp amplifier (Axopatch 200 A, Molecular Devices). Data were acquired using pClamp 10.0 software (Molecular Devices), filtered at 2 kHz, digitized (10 kHz), and analyzed offline using Clampfit10 (Molecular Devices). For spontaneous and evoked EPSC recordings, patch pipettes (3–5 MΩ) were filled with intracellular solution containing (in mM): Cs-methane sulfonate 135, HEPES 10, MgATP 2, NaGTP 0.3, CaCl_2_ 0.4, MgCl_2_ 2, QX-314 2, and BAPTA 5 (pH adjusted to 7.3 with CsOH). In order to block GABAA receptors, 10 μM Bicuculline methochloride was added to the extracellular solution. Stimulation electrodes used to evoke EPSCs were placed inside a theta glass tube and filled with ACSF (tip 15–20 μm), positioned onto a manual manipulator connected to the unit of stimulation (Iso-stim A320, WPI) to control the quantity of the current applied to stimulate the presynaptic fibers. The stimulation electrode was placed in the *stratum radiatum* (around 80 µm toward CA2), to activate the Schaffer collaterals projecting to CA1 neurons. To obtain the input/output curves (I/O), Schaffer collaterals were stimulated with currents of increasing intensity (0.1, 0.5, 1, 3, 7, 10 mA), holding the potential at −70 mV, to observe the AMPAR-mediated responses. Each stimulus lasted for 0.1 ms and was given 6 times, one every 10 s. The amplitude of around 6 responses for each stimulation was then averaged to obtain the I/O curve. 

Patch clamp recordings of CTRL and ABX microglia were performed in whole cell configuration exploiting the GFP expression by microglial cells, in the CA1 stratum radiatum at 50 μm under the slice surface, in order to avoid potentially activated microglia by the slicing procedure. Moreover, experiments were performed from 1 to 7 h after slicing at room temperature. Slices were perfused with ACSF as already described. The ACSF was continuously oxygenated with 95% O_2_, 5% CO_2_ to maintain physiological pH. Patch pipettes (4–5 MΩ) were filled with an intracellular solution containing the following composition (in mM): KCl 135, EGTA 0.5, MgCl_2_ 2, CaCl_2_ 0.011, HEPES 10 e Mg-ATP 2 (pH 7.3 adjusted with KOH, osmolarity 290 mOsm; Sigma Aldrich). Voltage-clamp recordings were performed using an AxonMulticlamp 700B (Molecular Devices, LLC, Sunnyvale, CA, USA). Currents were filtered at 2 kHz, digitized (10 kHz) and collected using Clampex 10 (Molecular Devices); the analysis was performed off-line using Clampfit 10 (Molecular Devices). To determine the current/voltage (I/V) relationship of each cell, voltage steps from −170 to +70 mV (V = 10 mV) for 50 ms were applied, holding the cell at −70 mV between steps. Resting membrane potential and membrane capacitance were measured at the start of recording. Data of both outward and inward rectifier K+ current amplitude were assessed after subtraction of the leak current by a linear fit of the I/V curve between −100 and −50 mV. Only cells whose current showed a rectification above −30 mV and the amplitude measured at 0 mV was at least 10 pA, after leak subtraction, were considered as expressing the outward rectifier K+ current; similarly, cells which showed a small inward rectification below −100 mV were classified as expressing the inward rectifier K+ current when subtracted current amplitude was at least 5 pA at −150 mV. 

### 2.3. Time-Lapse Imaging

The rearrangement of microglia processes towards a local injection of ATP [31] was evaluated on acute hippocampal slices acquiring time-lapse images, after at least 2 h of recovery. Slices were constantly kept in oxygenated ACSF during all the stages of the experiment at room temperature. Images were acquired every 10 s for 50 min, (exposure time of 200 ms) using a BX51WI microscope (Olympus Corporation, Tokyo, JP equipped with two objectives: LUMPlanF N 10×/0.10, air, and 40×/0.80, water immersion, Olympus Corporation). An Optoscan monochromator (Carin Research, Facersham, UK) was used to excite the GFP at 488 nm. Light was generated by a xenon lamp Optosource (Cairn Research). A micropipette of borosilicated glass was filled with ACSF supplemented with Mg-ATP 2 mM (Sigma Aldrich), and moved via a micromanipulator MP-225 (Sutter Instruments, Novato, CA, USA) to reach the core of the field recording, around 50 µm beneath the surface of the slice. The basal fluorescence was assessed for 5 min, then a small volume of Mg-ATP solution was puffed at the core of recording field via a pneumatic pico-pump (PV820; World Precision Instruments, Inc., Sarasota, FL, USA) with a brief pressure (8 psi; 100 ms). The images, collected with a camera CCD CoolSnap MYO (Photometrics, Tucson, AZ, USA), were analyzed using MetaFluor software as fluorescence variation measured into five concentric circular regions (regions of interest, ROI) positioned from the tip of the ATP pipette, with a diameter of 10, 20, 40, 80, and 120 µm. To determine the signal, the formula (F-F0)/F0 was used, where F0 matches the average fluorescence before the ATP application and F refers to the average fluorescence after the ATP application. To find out the effect of ABX treatment on microglia processes recruitment, we evaluated the increase in fluorescence in concentric regions of interest positioned around the tip of the ATP-containing pipette [30,32]. Measurements were collected after a brief puff of ATP (2 mM, 100 ms) in hippocampal slices from CTRL and ABX-treated mice.

### 2.4. Tracking Analysis of Microglia Dynamics

Microglia basal motility was observed taking advantage of an upright microscope (Olympus BX51WI) with a 40 × 0.8 NA water immersion objective. GFP was excited at 488 nm, with a 150 W lamp and a monochromator. Stack images were acquired at room temperature (24–25 °C), on slices perfused with oxygenated ACSF for 30 frames at 0.1 frames/sec, using a CoolSnap Myo camera and MetaFluor software (Molecular Devices, Foster City, CA, USA). Microglia dynamics were, thus, tracked using ImageJ processing package Fiji and the tracking plugin MTrackJ, as previously reported [30]. Once tracked, a custom-written script, implemented in Matlab, was used for correcting the minimum spatial resolution of the tracks within the acquired image [26]. 

In brief, tracks were traced and transferred into a new coordinate system in which the origin (x = 0, y = 0) was set as the starting position of each process. Consequently, the tracks were processed with the Matlab script in order that the distance of each point of tracking was set to the minimum resolution distance between the points (i.e., d = 0.61 * wavelength/numerical aperture = 0.37 mm). Tracks that endured more than 2 min with a distance less than 2d were excluded. The customized tracks obtained were subsequently analyzed to obtain the displacement, length, and instantaneous speed.

### 2.5. Morphology and Microglia Density Analysis

Cx3cr1^+/gfp^ and Cx3cr1^gfp/gfp^ mice were transcardially perfused with PBS and 4% PFA; whole brains were maintained in 4% PFA overnight and then incubated in 30% sucrose PBS solution overnight at 4 °C. Brains were stored at −80 °C until sectioning. Frozen brains were cut into 50-µm-thick horizontal slices (Leica cryostat) and stored at 4° until use. Images from Cx3cr1^+/gfp^ and Cx3cr1^gfp/gfp^ mouse slices were acquired exploiting a Nikon Eclipse Ti equipped with X-Light V2 spinning disk (CrestOptics), LDI laser source (89 North) and Prime BSI Scientific CMOS (sCMOS) camera, 6.5 µm pixels (Photometrics) with a 10×/0.25 Plan E air objective and a 60×/1.4 PlanApo l oil objective. Moreover, Metamorph software version 7.10.2 (Molecular Devices) was used to acquire GFP signal with a step size of 3 µm (for 10×) and 0.1–0.3 µm (for 60×). Through ImageJ software, maximal intensity z projections were obtained in order to get representative images of the acquired fields. All the cells that appeared entire within each acquired stack were subjected to the analysis. Microglia density was evaluated by counting the number of cell bodies totally contained within the z-projection, taking advantage of the endogenous GFP expression. The obtained number was normalized on the acquired volume in each acquisition field. For microglia morphometrical analysis, all the entirely visible cells inside the acquisition field were analyzed. Cells were then skeletonized on the binary images, using the ImageJ dedicated plug-in.

### 2.6. Dendritic Spine Density Analysis

Dendritic spine density analysis in the hippocampal stratum radiatum was performed from 60-µm-thick coronal brain slices of Thy1::EGFP-M21 perfused mice. Images were acquired as previously described, using a 100× PlanApo l oil objective (1.45 numerical aperture). The slices in Z were sliced with a step size of 0.1 µm. Signal deconvolution was applied through Huygens software (Huygens professional, Scientific Volume Imaging).

The analysis was performed on secondary and tertiary dendrites starting from maximum z-projection of the planes containing the dendrite segment of interest (ImageJ software). Four dendritic segments were randomly chosen in the field of view (2 fields per slice, six slices per mice, two mice for each condition). The dendrite was then reconstructed and measured to evaluate neurite spine density using NeuronStudio software (version 0.9.92 64-bit, Computational Neurobiology and Imaging Center Mount Sinai School of Medicine, New York, NY, USA).

### 2.7. Real Time PCR

Total RNA was extracted from hippocampal tissue with the Quick RNA MiniPrep (Zymo Research, Freiburg, DE) and retro transcribed with iScript Reverse Transcription Supermix for Real-time PCR (RT-PCR) (Bio-Rad, Hercules, CA, USA). RT-PCR was carried out using Sybr Green (Biorad) according to the manufacturer’s instructions. The PCR protocol consisted of 40 cycles of denaturation at 95 °C for 30 s and annealing/extension at 60 °C for 30 s. For quantification analysis the comparative Threshold Cycle (Ct) method was used. The Ct values from each gene were normalized to the Ct value of GAPDH in the same RNA samples. Relative quantification was performed using the 2^−∆∆Ct^ method (Schmittgen and Livak, 2008) and expressed as fold change in arbitrary values. Primer sequences targeted against GAPDH forw: TCG TCC CGT AGA CAA AAT GG, GAPDH rew: TTG AGG TCA ATG AAG GGG TC; P2Y12 forw CCT GTC GTC AGA GAC TAC AAG, P2Y12 rew GGA TTT ACT GCG GAT CTG AAA G; P2Y6 forw ATC AGC TTC CTG CCT TTC C, P2Y6 rew CTG TGA GCC TCT GTA AGA GAG ATC G.

### 2.8. NanoString nCounter Gene Expression Assay and Data Analysis

Hippocampal hemispheres were isolated from CTRL and ABX-treated mice. Total RNA was extracted with the Quick RNA MiniPrep (Zymo Research, Freiburg, DE, USA). NanoString nCounter Inflammation panel assays were performed using 50 ng of purified RNA following manufacturer’s instructions (NanoString Technologies). Sample preparation and hybridization reactions were performed according to manufacturer’s instructions (NanoString Technologies). All hybridization reactions were incubated at 65 °C for a minimum of 16 h. Hybridized probes were purified and counted on the nCounter SPRINT Profiler (NanoString Technologies) following the manufacturer’s instructions. Data analysis was performed using the nSolver analysis software (NanoString Technologies) (https://www.nanostring.com/products/analysis-software/nsolver) and housekeeping genes were used for data normalization. In order to identify the differentially expressed genes (DEGs), those with an interquartile range (IQR) value that stood under the 10th percentile of the IQR value distribution were discarded from the datasets. The expression levels were compared between groups using the paired Wilcoxon rank-sum test on normalized and log2-transformed data. Genes with p-value < 0.05 and fold change > 1.5 were considered as DEGs. Data analysis of gene expression value was performed using R (version 3.6.2).

### 2.9. Statistical Analysis

Statistical analysis was performed using Prism 5.0 and Origin 6.0 software R (version 3.6.2) and SigmaPlot. Data were evaluated for normal distribution and represented in the figures as mean ± s.e.m. For each figure, n = the number of independent biological replicates. Neither samples nor animals were excluded from the analyses. Quantitative RT–PCR, electrophysiological recordings, and time-lapse experiments were replicated at least four times with similar results. Differences among more than two groups with only one variable were assessed using one-way ANOVA with Tukey’s or Sidak’s post hoc test. Comparisons from nanostring gene analysis were analyzed using paired Wilcoxon rank-sum test on normalized and log2-transformed data. Two-way ANOVA with Sidak’s post hoc test was used for comparisons of two or more groups with two variables. Significant differences emerging from the above tests are indicated in the figures by * *p* < 0.05, ** *p* < 0.01, *** *p* < 0.001, **** *p* < 0.0001. Notable non-significant differences are indicated in the figures by NS.

## 3. Results

### 3.1. ABX Treatment Increases Microglia Density in the Hippocampus without Affecting the Expression Level of Inflammation-Related Genes

To assess whether the alteration of intestinal microbiota due to oral treatment with non-absorbable ABX may impact microglia control of brain parenchyma homeostasis, we treated four-week-old male Cx3cr1^+/gfp^ mice with a mix of two non-absorbable antibiotics (ABX: Gentamicin and Vancomycin) in drinking water for two weeks. As recently described in a report from our laboratory, our protocol of ABX administration induced mild dysbiosis in treated mice, with an overall reduction in gut microbiota species diversity and alteration of family abundance in the caeca. Specifically, phylogenetic analysis showed increase of Burkholderiales families and reduction of the Prevotellaceae, Rikenellacaea, and Helicobacteraceae families [33]. In accordance, all mice treated with antibiotics used for the experiments showed an enlargement of the ceaca as macroscopic evidence of dysbiosis.

**Figure 1:**
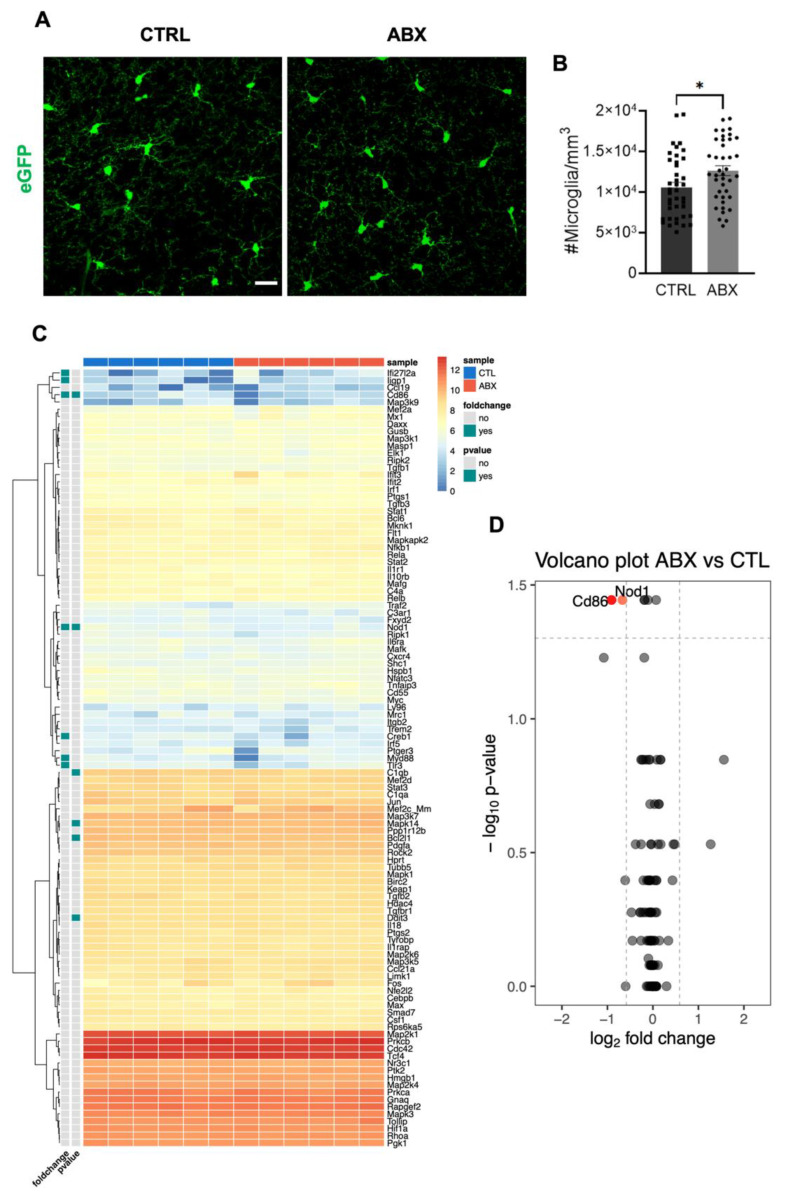
ABX treatment increases microglia density in the hippocampus with no variations in inflammatory gene expression in hippocampal parenchyma. ABX mice display a significant increase of hippocampal microglia density compared to control (CTRL) mice. (**A**) Representative z-stacks projection showing microglia cells in the hippocampal stratum radiatum of P40 Cx3cr1^+/gfp^ CTRL and ABX mice (Green= eGFP, scale bar 20 μm). (**B**) Bar chart showing microglia mean density in CTRL (10600 ± 600 GFP^+^ cells/mm^3^, n = 40 slices/6 mice, black) and ABX mice (12600 ± 600 GFP+ cells/mm^3^, n = 39 slices/6 mice, grey; * *p* < 0.05, Student’s t-test). (**C**) Heat map of unsupervised hierarchical clustering inflammation genes in CTRL (n = 6) and ABX hippocampus samples (n = 6) analyzed by NanoString nCounter gene expression assay. Colors in the heatmap indicate log2 counts normalized to housekeeping genes. Note that ABX treatment did not alter the expression of inflammatory-related genes. (**D**) Volcano plot showing the downregulation of Nod1 and CD86 in ABX hippocampus samples.

Confocal 3D scans of stratum radiatum of hippocampal slices from control and ABX-treated Cx3cr1^+/gfp^ mice showed increased microglia density in ABX-treated mice as the number of microglia cells in tissue volume (Figure 1A,B). 

To assess if ABX treatment might affect brain homeostasis, we analyzed the inflammatory state of brain parenchyma by nanocounter gene expression analysis of total hippocampal RNA extracts from six control and six ABX-treated mice and found that on control and ABX hippocampal samples only 107 over the 248 genes within the Inflammation mouse panel were expressed. Among these we did not find any upregulation in transcript expression as shown by the heat map (Figure 1C), thus indicating the absence of an inflammatory state in the hippocampus upon ABX treatment. Moreover, we observed downregulation of Nod1 and Cd86 transcripts, as depicted in the volcano plot (Figure 1D). These results suggest that ABX treatment, while inducing a significant change of microglia density, did not modify inflammation-related gene expression in brain parenchyma.

### 3.2. ABX Treatment Alters Microglia Functional Properties in Acute Hippocampal Slices

**Figure 2:**
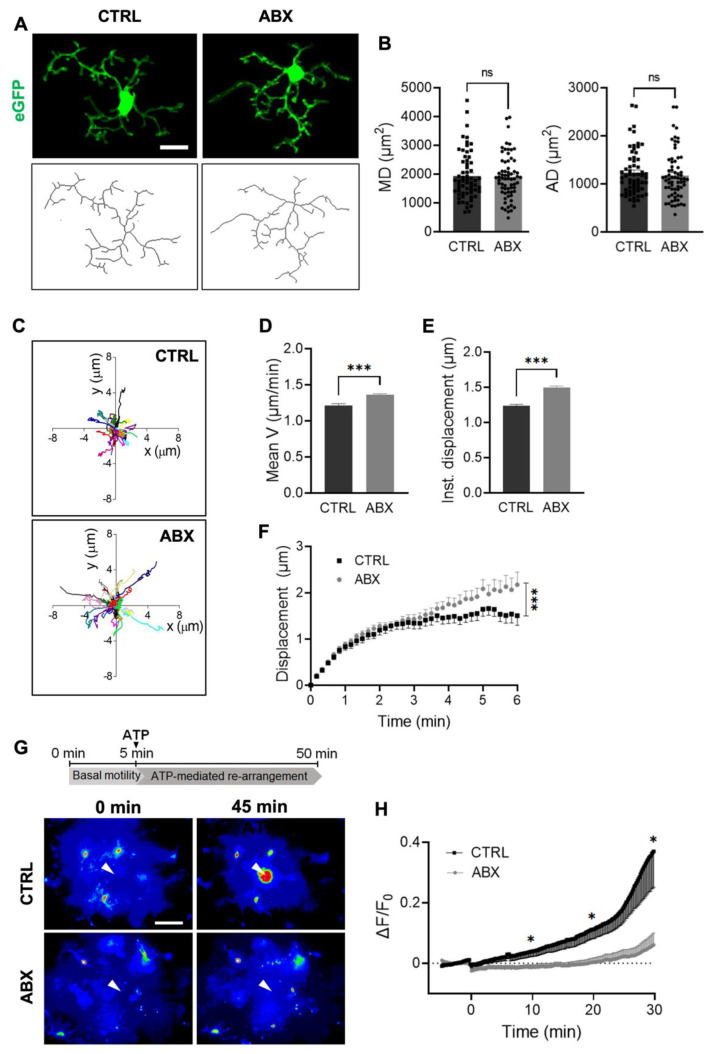
ABX treatment alters microglia functional properties in acute hippocampal slices. Quantitative morphometric analysis of microglia in CTRL and ABX mice. (**A**) Representative z-stacks projection showing GFP^+^ microglia (top) and tagged skeleton (bottom) of CTRL and ABX mice (scale bar 10 µm). (**B**) Bar charts of morphometric parameters: microglial domain (MD, left) is the area defined by the longest cell processes (CTRL n = 54 cells/ 5 mice; ABX n = 67 cells/5 mice, *p* = 0.8), while arborization domain (AD, right) is the area defined by all the cell processes, describing the overall arborization (CTRL n = 62 cells/5 mice, ABX n = 66 cells/5 mice, *p* = 0.52, Student’s t-test). (**C**) Track analysis of microglia processes basal motility in hippocampal slices from CTRL (left panel) and ABX-treated mice, measured by time-lapse fluorescence monitoring in acute hippocampal slices (15 min). Bar graphs representing spontaneous patrolling activity parameters obtained from track analysis: (d) ABX treatment increased (**D**) mean processes velocity and (**E**) instantaneous microglia processes displacement compared to CTRL (CTRL n = 22 cells/ 4 mice, ABX n=44 cells/ 3 mice; Student’s *t*-test *** *p* < 0.001). (**F**) Plot showing time course of instantaneous radial process displacement in slices from ABX (grey circles) and CTRL (dark squares) mice (two-way ANOVA *** *p* < 0.001). (**G**) Representative fields of GFP fluorescence measurements in slices from CTRL and ABX Cx3cr1^+/gfp^ mice at minute 0 and after 45 min of ATP perfusion. The arrow represents the tip of the ATP puff pipette. After 5 min of basal motility recordings, ATP is applied for 45 min (Mg-ATP 2 mM, 8 psi, 100 ms) as shown in the timeline of the experiment (top). (**H**) Time course of fluorescence ratio signal (ΔF/F0) measured in a circle (10 μm radius) centered on the tip of the ATP-containing pipette, from slices of CTRL and ABX-treated Cx3cr1^+/gfp^ mice. (CTRL: black, n = 12 fields/4 mice and ABX: grey, n = 12 fields /4mice; * *p* < 0.05, One-way ANOVA at minutes 10, 20 and 30). Note the fluorescence increase in the area around the pipette tip only in control slices. Scale bar: 20 μm.

We then analyzed the morpho-functional properties of hippocampal microglia in Cx3cr1^+/gfp^ mice treated with ABX. First, microglia morphology was assessed in confocal 3D scans of stratum radiatum of hippocampal slices from control and ABX mice, showing that the treatment-induced increase in density was not linked to changes of microglia morphology. Indeed, the analysis of several morphometric parameters of GFP-positive cells in stratum radiatum, obtained by the skeletonization of single microglia cells, showed that they were unaffected by ABX treatment (Figure 2A,B). 

In parallel, we analyzed by whole-cell patch clamp recording the electrophysiological properties of visually identified microglial cells within the stratum radiatum of acute hippocampal slices from Cx3cr1^+/gfp^ mice. Consistent with the lack of morphological changes, we observed that ABX treatment left unaltered the pattern of voltage-activated potassium currents recorded in patch clamped microglia (Supplementary Figure S1). 

To further investigate the impact of ABX treatment on microglia functions, we focused on patrolling activity, analyzing microglia processes movement in acute hippocampal slices in basal condition and in response to an ATP source. Tracking analysis (Figure 2C) of spontaneous microglia patrolling indicated that in slices from ABX mice, microglia constantly moved their processes with an increased mean velocity (Figure 2D); in addition, measurement of the instantaneous process displacement showed a higher processes displacement in microglia from ABX mice (Figure 2E). This is supported by the instantaneous process displacement plot (Figure 2F), representing how the displacement of the moving processes varies over time, showing that the time-dependent increase in radial distance was higher in hippocampal slices from ABX mice.

Microglia ability to extend processes towards the site of a local ATP application was assessed by time-lapse acquisition in hippocampal slices from Cx3cr1^+/gfp^ mice. This procedure typically gives rise to an increase in the fluorescence level around the pipette tip, due to the extension of microglia processes towards the ATP source. In hippocampal slices from ABX-treated mice we observed a significant reduction of the fluorescence increase around the pipette (20 μm radius area; Figure 2G,H), suggesting a reduced ability to respond to ATP. 

Real time PCR evaluation of purinergic receptors transcript levels on total hippocampal RNA extracts from control and ABX-treated mice revealed increased expression of p2y12 and p2y6 transcripts (see Supplementary Figure S2), as previously reported [33].

Taken together, these data indicate that ABX treatment increases microglia density and basal motility, likely favoring the homeostatic patrolling of hippocampal parenchyma. On the other hand, microglia from ABX-treated mice are unable to respond to purinergic damage signals.

### 3.3. ABX Treatment Impairs Hippocampal Synaptic Transmission

**Figure 3:**
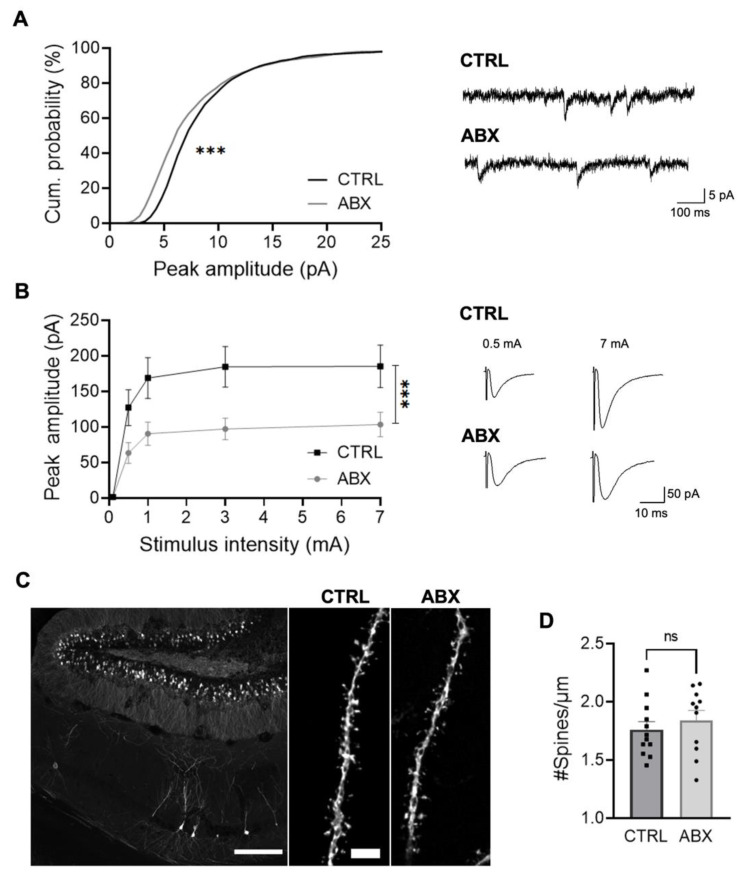
ABX treatment impairs hippocampal glutamatergic synaptic transmission in Cx3cr1^+/gfp^ mice. (**A**) *Left.* Cumulative distribution of sEPSCs recorded from Cx3cr1^+/gfp^ CA1 neurons at −70 mV; CTRL (CTRL mean peak amplitude 8.86 ± 0.3; n = 11 cells/4 mice, black) and ABX (ABX mean peak amplitude 8.05 ± 0.6; n = 14 cells/4 mice, grey). *Right.* Representative EPSCs recorded at −70 mV from CTRL and ABX mice. Note smaller peak amplitudes in ABX compared to CTRL mice (Kolmogorov-Smirnov test, *** *p* < 0.05). (**B**) *Left.* Plot showing the input–output curve of evoked EPSC peak amplitudes at CA3-CA1 synapses recorded at −70 mV from CTRL (n = 15 cells/3 mice, black) and ABX mice (n = 14 cells/4 mice, grey). *Right.* Representative traces of evoked EPSCs recorded at −70 mV from CTRL and ABX Cx3cr1^+/gfp^ CA1 hippocampal neurons in slices from CTRL and ABX mice at 0.5 and 7 mA stimulation. Note that in ABX-treated mice, neurons show significantly lower peak amplitudes compared to CTRL (*** *p* < 0.001, two-way ANOVA). (**C**) Representative confocal images of dendritic segments of CA1 pyramidal neurons in hippocampal slices from CTRL and ABX-treated Thy1::EGFP-M21 mice (scale bar: 200 μm; zoom scale bar: 3 μm). (**D**) Bar chart representing mean dendritic spine density in the two conditions (CTRL 1.76 ± 0.07 spines/μm, n = 12/2 slices/mice); ABX 1.84 ± 0.08 spines/μm, n = 11/2, Student’s t-test *p* = 0.45).

Considering the deep interplay between neuronal and microglial cells in the modulation of synaptic activity, we wondered whether ABX-induced functional changes in microglia could cause changes in synaptic properties. We assessed the excitatory synaptic transmission of CA1 pyramidal neurons in acute slices from control and ABX-treated Cx3cr1^+/gfp^ mice, by patch clamp recordings, in order to determine the impact of ABX treatment on hippocampal synaptic transmission. [26]. Recordings of CA1 pyramidal neurons from mice treated with ABX showed a significant decrease in the amplitude of spontaneous excitatory postsynaptic currents (sEPSC), compared to control, without major effects on sEPSC frequency (Figure 3A and Supplementary Figure S3A). Consistently, in ABX-treated mice, excitatory postsynaptic currents (EPSCs) evoked at CA3-CA1 synapses by Schaffer collaterals stimulation displayed strongly reduced amplitudes compared to control ones (Figure 3B). This is confirmed by the input/output curve, suggesting that ABX treatment deeply affects CA3-CA1 functional connectivity. 

To investigate if structural changes may underlie the observed reduced glutamatergic function, we evaluated the dendritic spine density in ABX-treated and CTRL mice. For this set of experiments we took advantage of the Thy1::EGFP-M21 mice, which express EGFP in sparse subsets of pyramidal neurons, providing a bright, vital Golgi-like staining, thus allowing dendritic confocal microscopy analysis of dendritic spines. Confocal 3D analysis of neuronal CA1 dendritic spine showed that the reduction of glutamatergic transmission was not associated with a change in spine density (Figure 3C,D). These results suggest that ABX treatment affects the synapse functionality, causing the weakening of glutamatergic synaptic transmission between Schaffer collaterals and CA1 pyramidal neurons, thus decreasing functional connectivity, without changes in the number of dendritic spines.

### 3.4. Microglia–Neuron Crosstalk through the CX3CL1/CX3CR1 Axis Is Required for the ABX Induced Reduction of Synaptic Transmission

To ascertain whether the effects induced by ABX treatment on glutamatergic synaptic transmission could be mediated by microglia–neuron crosstalk, we took advantage of a defective model of microglia–neuron interaction, based on the KO of the fractalkine receptor [26,30]. Indeed, in these mice, the lack of neuron–microglia crosstalk through the CX3CL1/CX3CR1 axis is known to delay synaptic maturation and connectivity [22,24,25,34,35].

**Figure 4:**
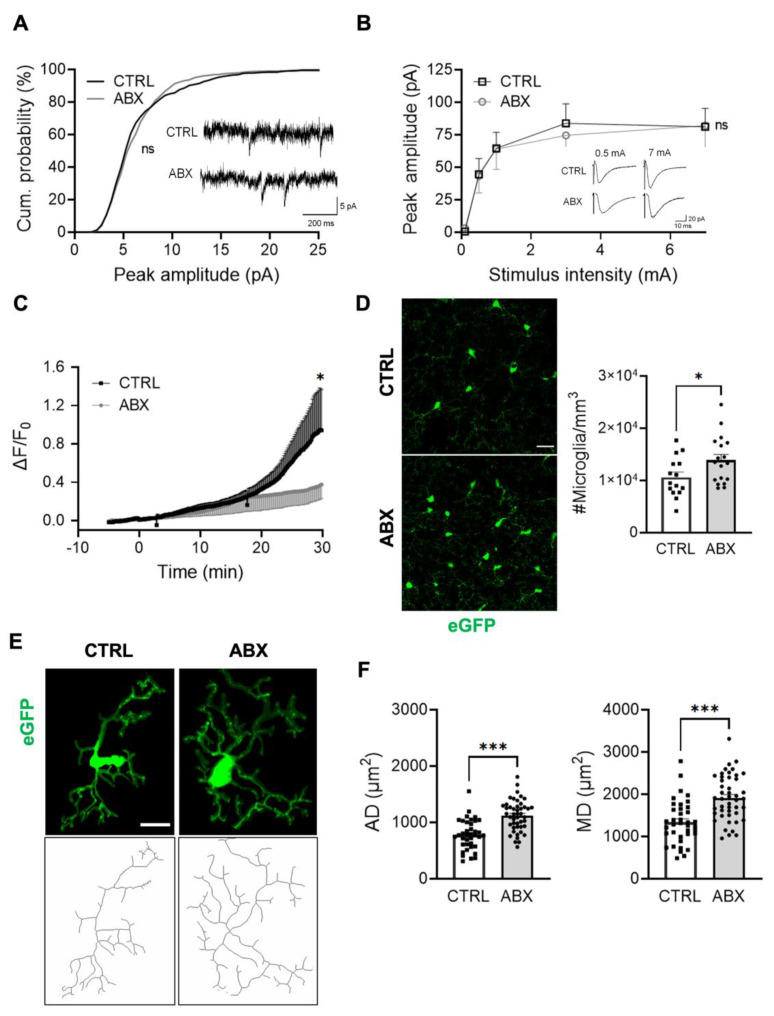
ABX-induced effects on synaptic transmission are absent in mice lacking CX3CR1. (**A**) Cumulative distribution of sEPSC current amplitude recorded from Cx3cr1^gfp/gfp^ CA1 pyramidal neurons (−70 mV holding potential) in slices from CTRL (mean peak amplitude 6.85 ± 0.1; n = 8 cells/3 mice, black) and ABX mice (mean peak amplitude 6.56 ± 0.1; n = 10 cells/3 mice, grey; Kolmogorov–Smirnov test, *p* = 0.18). Inserts: Representative traces of spontaneous EPSCs recorded at −70 mV. (**B**) Input–output curve of evoked EPSC peak amplitudes at CA3-CA1 synapses recorded at −70 mV from CTRL (n= 9/3, cells/mice, black) and ABX-treated Cx3cr1^gfp/gfp^ mice (n = 12/4, cells/mice, grey; two-way ANOVA; *p* = 0.86). Inserts: Sample traces of evoked EPSCs in CA1 pyramidal neurons from CTRL and ABX-treated Cx3cr1^gfp/gfp^ mice. Interestingly, in Cx3cr1^gfp/gfp^ mice ABX treatment left unaltered both spontaneous and evoked glutamatergic transmission. Note the reduced amplitudes of spontaneous (*p* < 0.01, *t*-test) and evoked EPSCs (two-way ANOVA; *p* < 0.05) in Cx3cr1 KO with respect to heterozygous mice. (**C**) Time course of fluorescence ratio (ΔF/F0), measured at ROI10 (10 μm radius centered on the tip of ATP containing pipette) after 2 mM Mg-ATP solution application (8 psi, 100 ms) on stratum radiatum of acute hippocampal slices from CTRL and ABX Cx3cr1^gfp/gfp^ mice (one-way ANOVA * *p* < 0.05, at minute 30). Note that ATP-mediated processes rearrangement in untreated mice is similar in both genotypes (one way ANOVA, Dunn’s multiple comparison test). (**D**) Left: Representative z-stacks projection of hippocampal stratum radiatum of Cx3cr1^gfp/gfp^ CTRL and ABX mice (scale bar 20 um). Right: bar chart of mean microglia cell density in both conditions (CTRL 9300 ± 700 cells/mm^3^; n = 15 fields/2 mice, black bar; ABX 11700 ±700 cells/mm^3^; n = 18 fields/2 mice, grey bar; Student’s t-test * *p* value < 0.05). (**E**) Representative z-stacks projection of GFP+ microglia (top) and tagged skeleton (bottom) of CTRL and ABX Cx3cr1^gfp/gfp^ mice. (**F**) Quantitative morphometric analysis of microglia from Cx3cr1^gfp/gfp^ CTRL and ABX mice. Left: bar chart of microglia arborization domain in CTRL (36 cells/10 fields/2mice) and ABX (46 cells/12 fields/2mice; Student’s t-test *** *p* < 0.001); Right: microglial domain in CTRL (n = 36 cells/10 fields/2 mice) and ABX (n = 46 cells/12 fields/2 mice; Student’s t-test *** *p* < 0.001).

It has to be noticed that, while the impairment of synaptic transmission due to the lack of CX3CL1/CX3CR1 signaling develops in the first postnatal weeks [24], and persists in the adult [22,26], the alteration of functional properties of microglia cells, such as ATP processes rearrangement, are only transiently present during the second and the third postnatal weeks and recover in adulthood [30], thus making this model suitable to dissect a possible role of microglia–neuron crosstalk in the ABX-induced impairment of glutamatergic synaptic transmission. We thus treated Cx3cr1^gfp/gfp^ mice with ABX for two weeks. Figure 4 shows that the absence of the CX3CL1/CX3CR1 axis prevented the modulation of synaptic transmission caused by ABX treatment.

Specifically, ABX treatment did not affect the amplitude as well as the frequency of spontaneous excitatory postsynaptic currents (sEPSC; Figure 4A and Supplementary Figure S3B). Moreover, when we analyzed the CA3-CA1 input/output curve, EPSCs displayed similar amplitudes in control and ABX-treated mice (Figure 4B), suggesting that the CX3CL1/CX3CR1 axis is required for the ABX effect on synaptic transmission.

Conversely, ABX treatment profoundly affected hippocampal microglia, reducing their ability to rearrange their processes towards locally applied ATP (Figure 4C), increasing microglia density (Figure 4D) and, noticeably, ramification (Figure 4E,F). In addition, tracking analysis of spontaneous microglia processes movement indicated that in slices from CX3CR1^gfp/gfp^ mice, ABX treatment reduced the mean velocity of microglia processes movement, leaving unaltered the instantaneous displacement (Supplementary Figure S4). 

Altogether, these data showing that ABX treatment altered microglia structural and functional characteristics in Cx3cr1 KO mice, leaving unaltered spontaneous and evoked EPSC, give rise to the idea that ABX effects on gut microbiota alter neuronal function through microglial dysfunction, thus pointing to a microbiota–microglia–neuronal axis.

## 4. Discussion

In this study we explored the impact of oral treatment with non-absorbable ABX on functional properties of hippocampal microglia cells and synaptic transmission. In particular, we analyzed the effect of chronic non-absorbable ABX treatment on basal and ATP-induced microglia processes motility and glutamatergic synaptic transmission in mouse acute hippocampal slices. Indeed, the modulation of these activities, specifically associated with the resolution of tissue damage and the activity of neuronal networks, may be relevant for the immunomodulatory role of microbiota–gut–brain axis on neuronal functions. 

Specifically, we report that non-absorbable ABX treatment (i) increases hippocampal microglia density, without affecting their morphology, (ii) changes the pattern of patrolling activity, and (iii) impairs the ability to rearrange processes in response to ATP. In addition, ABX treatment depresses hippocampal glutamatergic spontaneous and evoked synaptic transmission. Since microglial but not synaptic effects of ABX treatment are observed in mice lacking CX3CR1, we conclude that the ABX effects on glutamatergic synapses are mediated by the microglia–neuron crosstalk through the CX3CL1/CX3CR1 axis.

The modulation of microglia patrolling activity by host gut microbes has been demonstrated by a functional assay, monitoring microglia processes movement in basal conditions and in response to a local application of ATP, mimicking tissue damage [31]. In particular, in hippocampal slices from ABX-treated mice, we observed the alteration of basal patrolling activity and the impairment of ATP-induced processes motility. It has been widely reported that under physiological conditions, microglia continuously monitor brain parenchyma, through the extension and retraction of branches [36,37]. This activity is modified in the presence of an injury when, following ATP release by damaged neurons and the activation of purinergic receptors P2Y6 and P2Y12 [38,39], microglia rearrange their processes towards the site of damage [31,38,40,41]. 

Here, after two weeks of ABX administration, the ATP-mediated processes rearrangement [30,32] is significantly impaired, suggesting a reduced ability of microglia cells to start a rapid response to tissue damage. Microglia density and morphology as well as ATP sensitivity [30,32] are often involved in reduced ATP-mediated process attraction. However, the reported ABX effect cannot be ascribed to reduced ramification or downregulation of p2y12 transcript or protein [33], pointing to the involvement of an intermediate amplificatory step [31,42] or other control steps of either extracellular ATP degradation or the rearrangement process. Indeed the speed of ATP-mediated processes attraction may be influenced by amplificatory mechanisms, causing ATP release [43] as well as by the degradation of ATP by extracellular enzymes [44,45] and by the effects of the products of its catabolism (ADP, adenosine [46,47,48]). Finally, although, we cannot exclude a reduction of functionality of ATP receptors, other downstream membrane events could also be responsible for the reduction of the speed of processes movement [49,50].

On the other hand, we observed significant changes in the pattern of basal processes motility in slices from ABX-treated mice. Specifically, we report an increase of processes displacement and mean velocity, suggesting a larger scanning territory. Based on these data, we speculate that the ABX treatment could improve the ability of microglia processes to sample the surrounding brain parenchyma. However, these changes were not associated with an increase in cell ramification, but only in cell density. This seems to be in contrast with recent reports in germ-free or microbiota-depleted SPF adult mice, where microglia displayed an immature and hyper-ramified phenotype [17,51]. However, it has to be noted, that our experimental protocol, based on two weeks of administration of two non-absorbable antibiotics, does not eradicate gut bacteria [33]. Consistently, nanostring analysis of hippocampal extracts did not show changes in the transcript level of inflammatory genes denoting a relatively mild treatment. Indeed, we found the downregulation of only 2 over 248 genes, Nod1 and CD86, not allowing gene ontology analysis. However, Nod1 and CD86 may play a role in the gut–brain axis. Indeed, the expression of Nod1 had been recently reported to regulate central and peripheral serotonergic biology, and thus to be related to the proper function of gut–brain axis signaling in mice [52]. Moreover, decreased CD86 gene expression has been reported in microglia isolated from germ-free mice [17]. 

We speculate that the alterations of microglia patrolling properties might arise from a change in the pattern of the tissue molecular cues, like signals from gut bacteria, that are necessary for proper microglial functions [32,51,53,54]. Possible candidates are short-chain fatty acids (SCFA), gut bacteria metabolites crossing the gut barrier. SCFAs are reduced in germ-free and ABX-treated mice, and are able to rescue microglia phenotype in germ-free mice [17].

The use of ABX to manipulate microbiota confers an experimental advantage representing a tool to model the clinical scenario in humans and allowing us to determine the effect of such treatments on brain functions. ABX treatment offers much greater temporal flexibility and specificity compared to the GF model of microbiota ablation, as ABX can be delivered acutely or chronically at any life stage. Moreover, the appropriate choice of the ABX composition and dosage allows for a greater level of control over the extent of microbiota alteration, from minor perturbations through subtherapeutic doses of a single antibiotic, to entire microbiota ablation by specific ABX cocktail design. It has to be noticed that, in respect to the protocol established in Erny et al. (2015), we used only two out of four oral non-absorbable antibiotics at lower doses (vancomycin and gentamicin 0.5 mg/mL instead of 1 mg/mL) and for shorter time (two weeks instead of four), specifically to avoid the complete eradication of the intestinal microbiota, as shown in D’Alessandro et al. (2020). In particular, vancomycin, the drug of choice for gastrointestinal diseases, was associated with gentamicin, to get rid of the potential development of vancomycin-resistant enterococci [55,56,57]

An important consideration in the use of ABX to investigate the MGBA axis is their systemic entering from the gut. Non-absorbable ABX (i.e., vancomycin, neomycin, and gentamicin), which do not enter the systemic circulation, can be used to manipulate gut microbiota, avoiding potential systemic and CNS effects and thus allowing the direct assessment of MGBA. Conversely, ABX that can potentially enter the CNS, such as metronidazole and minocycline, can have direct action on brain and behavior (e.g., the reduction of microglia pro-inflammatory mediators by minocycline) [11,58,59].

Notably, we report that the impact of a 2-week-long ABX treatment was not confined to microglia cells. Indeed, in ABX mice we found a functional impairment of adult glutamatergic CA1 synaptic function, as revealed by the reduction of the amplitudes of evoked and spontaneous EPSC. In particular, we observed a reduced efficacy in CA1 glutamatergic synapses, without a change in spine number, pointing to a functional reduction of glutamatergic synaptic transmission.

We also report that ABX treatment, while affecting structural and functional properties of microglia, did not produce any significant effect on synaptic properties of mice lacking the fractalkine receptor (Cx3cr1^gfp/gfp^ mice), a well-assessed model of dysfunctional neuron–microglia signaling, that displays reduced functionality of glutamatergic hippocampal transmission [22,24,25,26]. 

It has to be noticed that the effect of ABX treatment on the patrolling activity of hippocampal microglia in Cx3cr1^gfp/gfp^ mice, did not reproduce that observed in Cx3cr1^+/gfp^ mice. However, when interpreting these results, we have to take into account that the basal motility of microglia processes differs between the two genotypes. Indeed, in control condition, Cx3cr1^gfp/gfp^ microglia display higher mean velocity and higher instantaneous displacement (Supplementary Figure S5) in respect to Cx3cr1^+/gfp^, in accordance with Basilico et al. (2019); this could be ascribable to differences in sampling efficacy arising from lower arborization domain in Cx3cr1^gfp/gfp^ mice [26]. Thus, the reduction in microglia processes motility caused by ABX treatment in Cx3cr1^gfp/gfp^ mice can be explained by a reduction of the available patrolling area, due to the increased cell density and the larger arborization domain acquired by these cells [36]. These results also highlight the key role of CX3CR1 in microglia functional changes induced by gut dysbiosis. 

Concerning synaptic regulation, we speculate that the absence of effects in Cx3cr1^gfp/gfp^ mice is due to the overlap of the CX3CL1/CX3CR1 axis dysfunction with the ABX effect; indeed, synaptic currents are smaller in Cx3cr1 KO mice [23,24]. However, we would rule out a possible floor effect, despite the observed difference in EPCS amplitudes, since glutamatergic currents be further reduced inducing, for instance, long-term depression in these mice [24]. Thus, we consider the most conservative interpretation of these data, that ABX effects on glutamatergic EPSC rely on microglia–neuron crosstalk. This is also in line with the data obtained in a model of pharmacological depletion of microglia, where after PLX5622 (CSF1R inhibitor) administration, the properties of hippocampal CA1 synapses closely resemble those observed in Cx3cr1^gfp/gfp^ mice [35]. Indeed, PLX treatment did not produce synaptic depression in mice lacking CX3CR1, indicating an occlusion effect between microglia removal and dysfunctional neuron–microglia signaling [26]. Still, it has to be considered also the possibility that the lack of ABX effects could be due to other phenotypic features of the Cx3cr1 KO mice, which include differences in basal hippocampal synaptic properties. On the other hand, the report of a gene dose-dependent phenotype [23] raises the possibility that Cx3cr1^+/−^ mice represent an intermediate phenotype leading to an underestimation of ABX effects. 

## 5. Conclusions

In conclusion, our study highlights the importance of microglia in mediating the gut–brain axis control of synaptic functioning in the adult hippocampus. ABX-induced microbiota alteration impairs microglia control of brain parenchyma homeostasis and reduces the efficacy of glutamatergic synaptic transmission. Furthermore, the lack of ABX impairment of synaptic transmission in Cx3cr1 KO mice point to a pivotal role of microglia as a mediator in gut neuronal signaling in MGBA.

## Data Availability

The data that support the findings of this study are available from the corresponding author upon reasonable request.

## Supplementary Materials

The following are available online at https://www.mdpi.com/article/10.3390/cells10102648/s1, Figure S1: Voltage-activated potassium currents in microglia from control and ABX mice. Figure S2: Real time PCR analysis of microglia transcripts. Figure S3: Plots of the effect of ABX treatment on the interevent interval of sEPSCs from Cx3cr1^+/gfp^ and Cx3cr1^gfp/gfp^ mice. Figure S4: Tracking analysis of basal processes motility of microglia in CTRL and ABX-treated Cx3cr1^gfp/gfp^ mice. Figure S5: Bar charts representing comparison of morphological and dynamical parameters of microglia from Cx3cr1^+/gfp^ and Cx3cr1^gfp/gfp^ mice.
