# Supplementary figures and images for: Antibiotics Treatment Modulates Microglia–Synapses Interaction
Source: Cells. 2021 Oct 4;10(10):2648. doi: 10.3390/cells10102648 (PMC8534187; doi:10.3390/cells10102648)

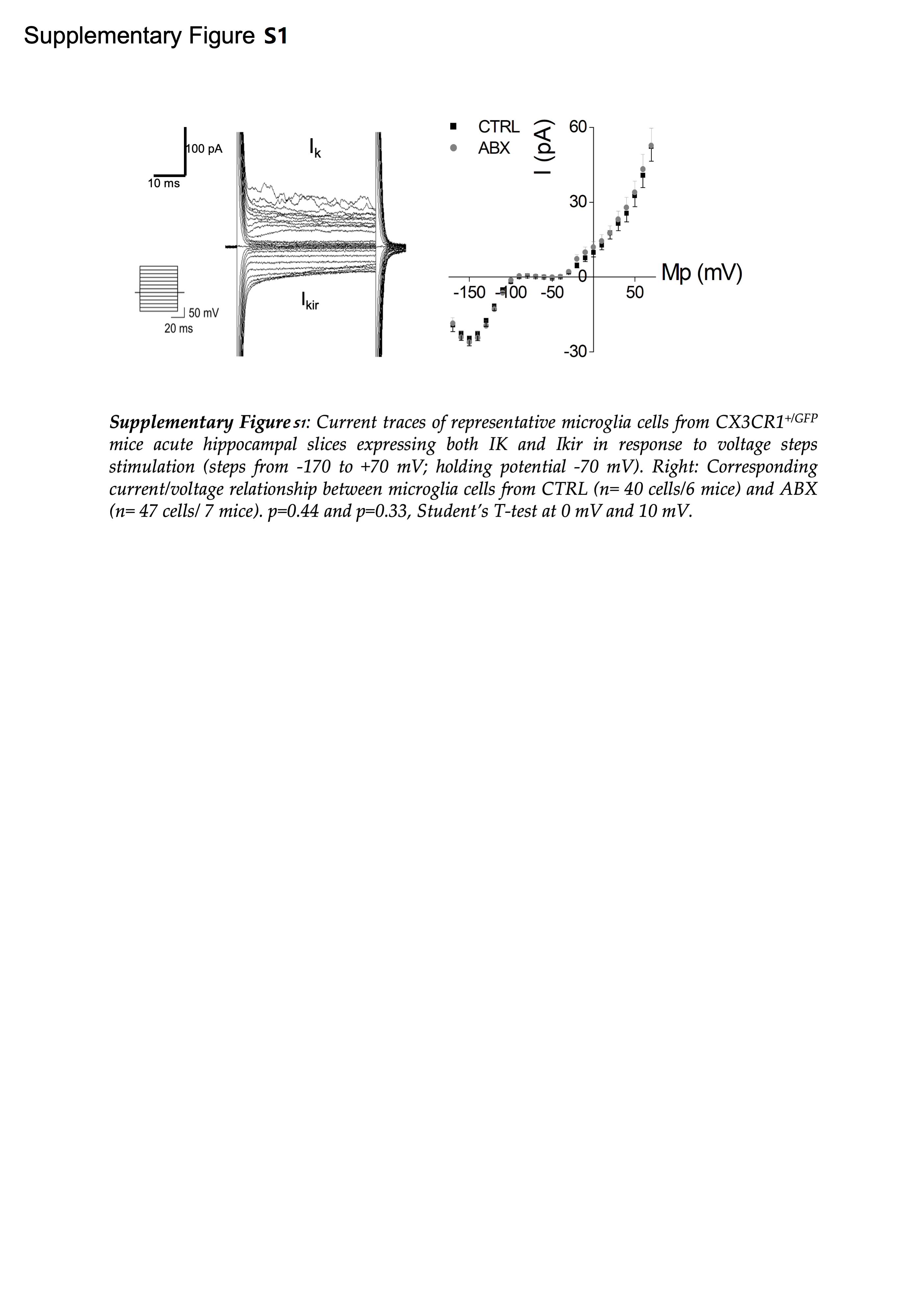

Supplement: Supplementary file 1 [file cells-10-02648-s001.zip › Supplementary Figure S1.tiff]

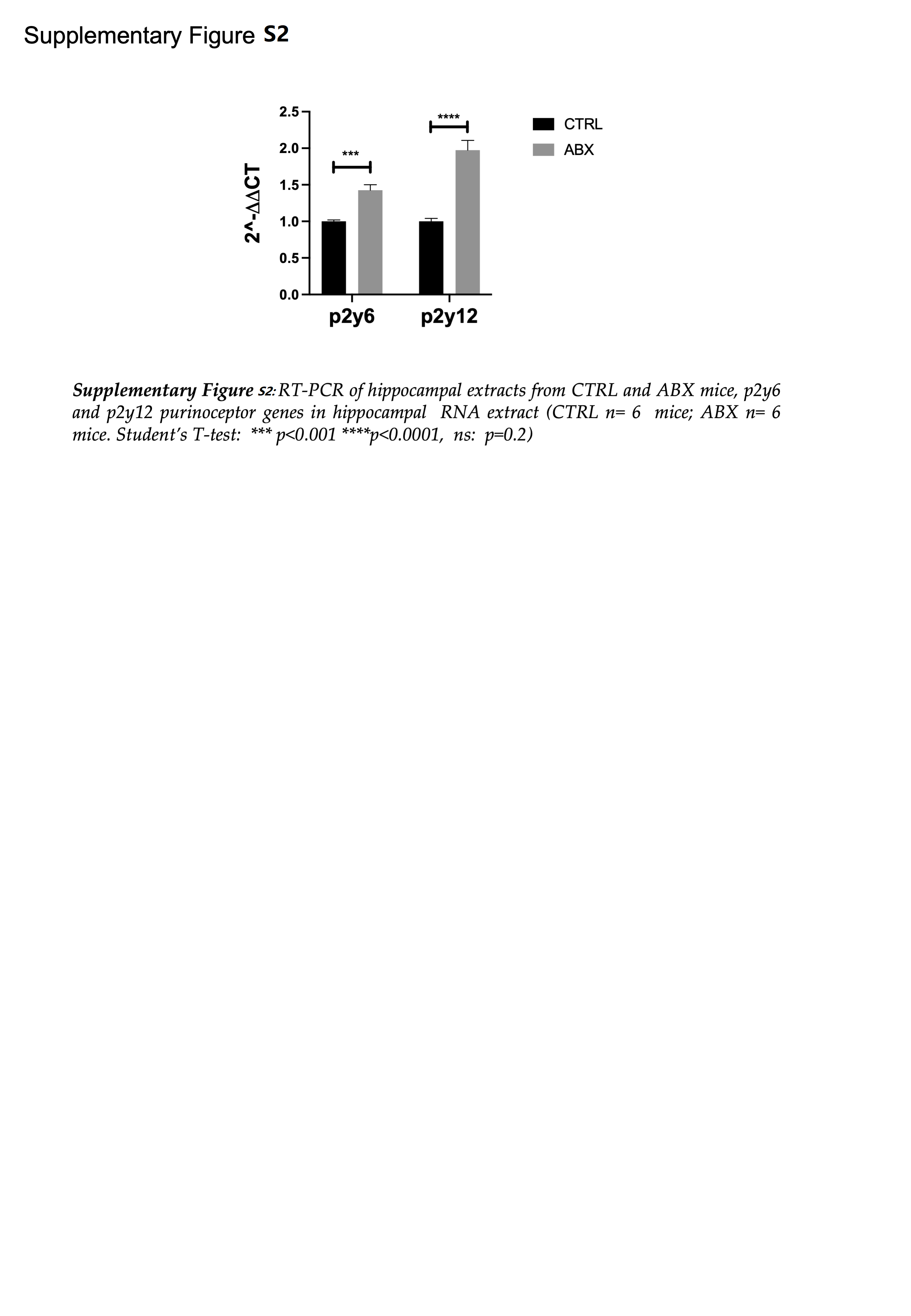

Supplement: Supplementary file 1 [file cells-10-02648-s001.zip › Supplementary Figure S2.tiff]

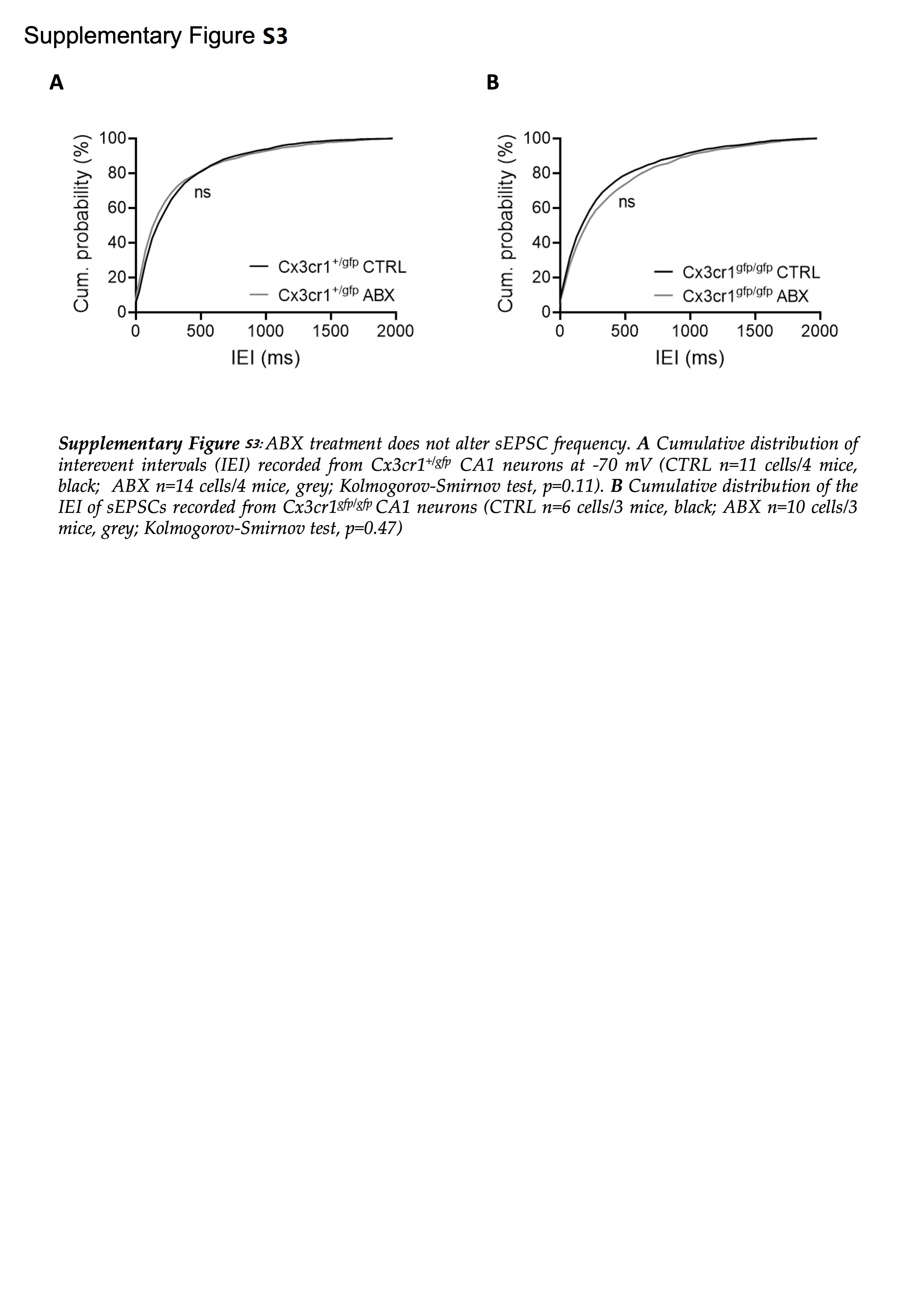

Supplement: Supplementary file 1 [file cells-10-02648-s001.zip › Supplementary Figure S3.tiff]

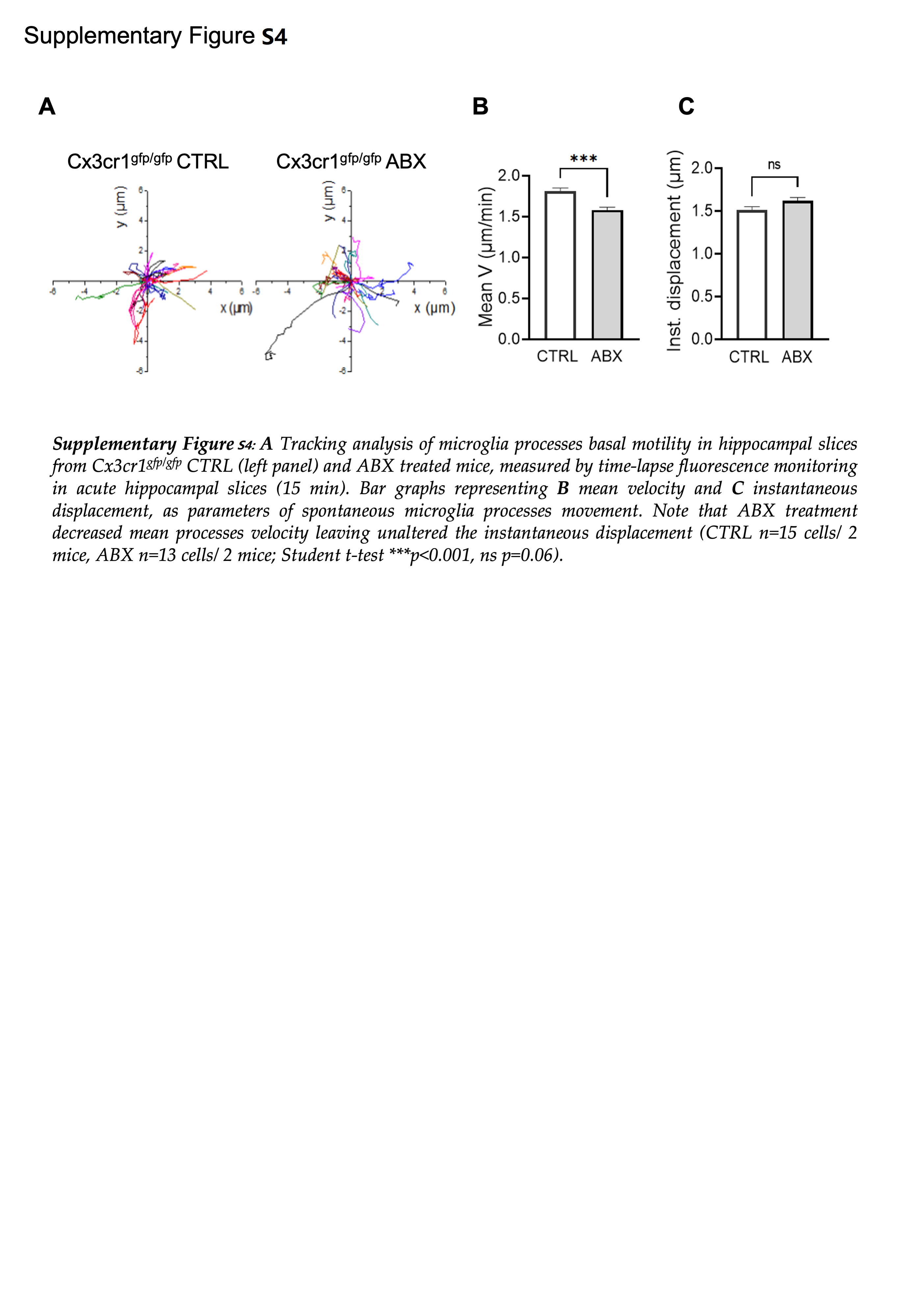

Supplement: Supplementary file 1 [file cells-10-02648-s001.zip › Supplementary Figure S4.tiff]

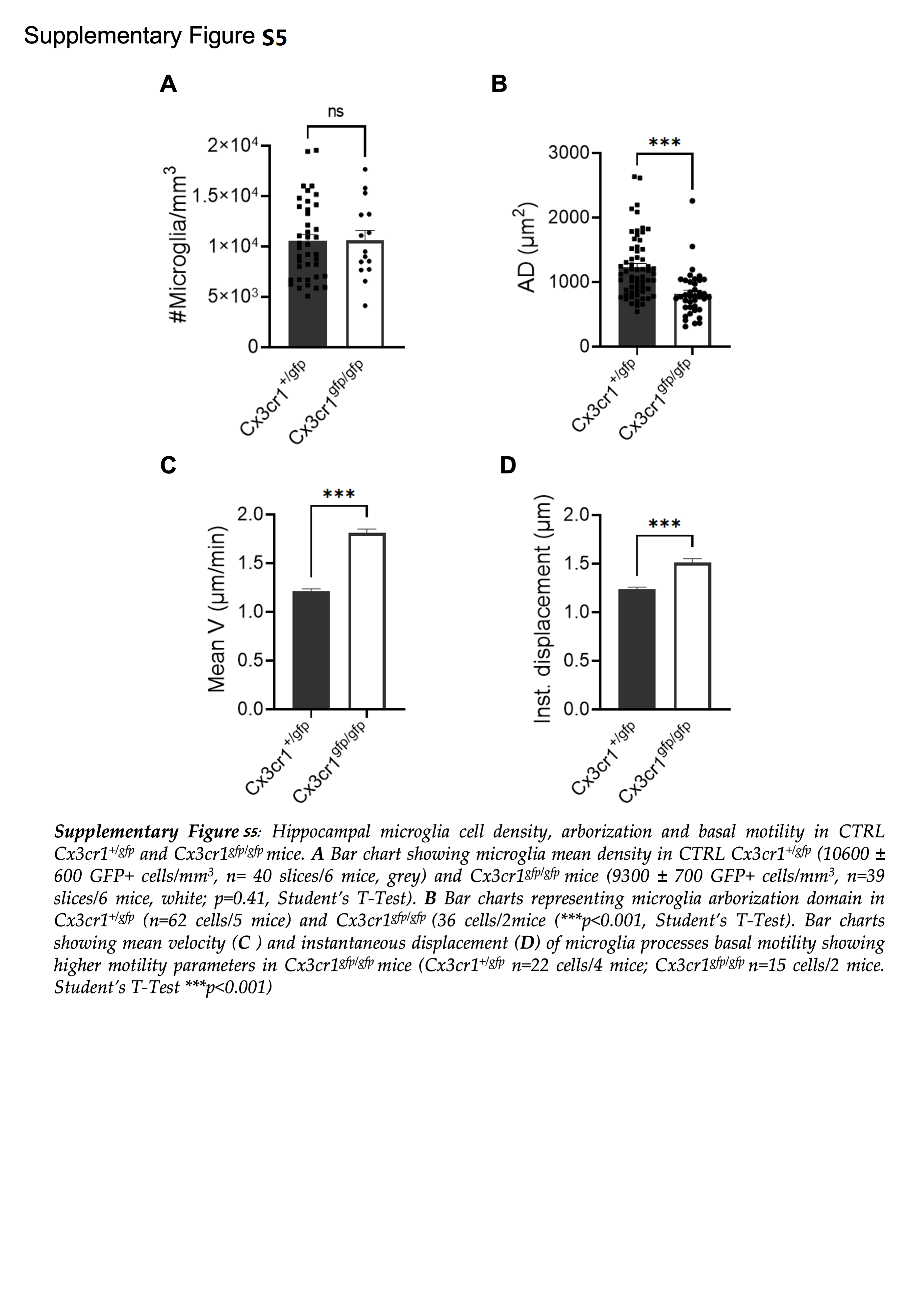

Supplement: Supplementary file 1 [file cells-10-02648-s001.zip › Supplementary Figure S5.tiff]
